# Do Histology and Primary Tumor Location Influence Metastatic Patterns in Bladder Cancer?

**DOI:** 10.3390/curroncol30100656

**Published:** 2023-10-11

**Authors:** Hyung Kyu Park

**Affiliations:** Department of Pathology, Chungnam National University School of Medicine, Daejeon 35015, Republic of Korea; hkpark@g.cnu.ac.kr

**Keywords:** bladder cancer, transitional cell carcinoma, metastasis, squamous cell carcinoma, adenocarcinoma, neuroendocrine carcinoma

## Abstract

Metastasis is the leading cause of death in patients with bladder cancer. This study utilized a statistical analysis of patient data from the Surveillance, Epidemiology, and End Results database to examine the influence of histological type and primary site on the metastatic behavior of bladder cancer. Significantly different metastatic patterns were observed among bladder cancer patients depending on their histological type. Patients with squamous cell carcinoma showed a significantly (*p* < 0.001) lower bone metastasis rate (27.2%) than patients with urothelial carcinoma (UC) (38.3%). Patients with neuroendocrine carcinoma showed a significantly (*p* < 0.001) higher liver metastasis rate (52.1%) and a significantly (*p* = 0.001) lower lung metastasis rate (25.7%) than patients with UC (22.6% and 33.5%, respectively). UC patients also demonstrated differences in metastatic behavior according to histological subtype. The sarcomatoid subtype showed a significantly (*p* < 0.001) higher lung metastasis rate (51.6%) and a significantly lower (*p* = 0.002) lymph node metastasis rate (22.6%) than the micropapillary subtype (12.1% and 54.1%, respectively). Significant differences in metastatic behavior were also observed among patients with conventional UCs originating from the bladder, ureter, and renal pelvis. This study highlights the impact of histological characteristics and primary site on metastatic tendencies in bladder cancer, highlighting the importance of tailoring treatment and surveillance strategies.

## 1. Introduction

Bladder cancer is the tenth most common cancer worldwide, with over 500,000 cases diagnosed in 2020, accounting for 3.0% of all newly diagnosed cancer cases globally [1]. The incidence of bladder cancer is steadily increasing, particularly in developed countries [2]. It is not clear why the incidence of bladder cancer continues to rise, especially in developed countries. However, it is thought to be influenced by an aging population, environmental factors, ongoing smoking rates, poor lifestyles, chemical exposures, and improved detection methods [2,3]. Bladder cancers can be histologically divided into urothelial and non-urothelial carcinomas [4,5]. Non-urothelial carcinomas are relatively uncommon, comprising only 10–20% of all bladder cancers, including squamous cell carcinoma (SCC), adenocarcinoma (ADC), neuroendocrine carcinoma (NEC), and other carcinomas. The remaining 80–90% of bladder cancers are urothelial carcinomas (UCs) [2].

UC arises from the urothelial cells, which are specialized cells that line the inside of the urinary tract and encompass the bladder, ureters, and renal pelvis. Thus, UC can also occur in the ureter or renal pelvis, where the urothelium is present [6]. However, it is more common in the bladder and accounts for approximately 90% of all UC cases. This discrepancy is likely attributable to variations in the mucosal surface area covered by the urothelium, carcinogen exposure, and genetic factors [7].

UC is a histopathologically heterogeneous cancer, with a high frequency of divergent differentiation [8]. There are many different histological subtypes of UC, with 13 histological subtypes recognized in the current version of the World Health Organization (WHO) classification [7,9]. The clinical significance of these histological subtypes has been widely studied and is presently controversial, with the majority of reports indicating that at least some variants are poor prognostic factors [8,9,10,11].

Mortality rates among patients with bladder cancer have decreased in recent years, particularly in developed nations, owing to medical advancements [12]. However, bladder cancer remains the cause of over 200,000 deaths worldwide, accounting for 2.1% of all cancer-related deaths worldwide [1]. Bladder cancer is still the ninth leading cause of cancer death in men worldwide. Metastasis is one of the most important factors affecting the five-year survival rate of patients with bladder cancer. While the five-year relative survival rate for patients with bladder cancer confined to the primary site was 70.9%, the corresponding rate for those with metastases decreased to 8.3% [13]. As the presence or absence of metastases has a remarkable effect on patient prognosis, researchers have focused on predicting metastatic behavior in patients with bladder cancer [14,15,16,17,18,19,20].

According to recent studies conducted in other organs, the patterns of tumor metastasis exhibit distinct variations depending on the histological type of the tumor [21,22]. Unfortunately, to the best of our knowledge, no studies have analyzed the differences in metastatic patterns based on histological type in patients with bladder cancer. However, several previous studies reported that bladder cancer shows clinical variations depending on its histological type [4,11,23,24,25]. Furthermore, a recent study analyzing gene expression in bladder cancer showed that UC has different gene expression patterns, depending on the molecular or histological subtype [26]. Some studies have reported differences in the molecular characteristics of UC depending on the primary tumor site [27,28,29]. And recently, microRNAs have been emerging as valuable biomarkers for diagnosing bladder cancer and predicting prognosis [30].

If there are differences in the clinical behavior and molecular characteristics according to the histological subtype and primary site of bladder cancer, it is likely that there are also differences in metastatic behavior. This study aimed to investigate the differences in metastatic tendencies among different histological types of bladder cancer, including UC, SCC, ADC, and NEC, the differences in metastatic tendencies among the histological subtypes of UC, and the differences in metastatic patterns based on the primary site of UC. To achieve this goal, data were extracted from the database provided by the Surveillance, Epidemiology, and End Results (SEER) program and subjected to comparative analysis.

## 2. Materials and Methods

### 2.1. Patient Data Extraction

A list of patients matching specific criteria was searched and extracted from the SEER database using the Case Listing Session in SEER*Stat software version 8.4.2 to perform statistical analysis [31]. The “Incidence—SEER Research Data, 17 Registries, Nov 2022 Sub (2000–2020)” database was selected from the several databases provided by the SEER program because it is the most current database with data from 17 registries and is the largest number database [32]. This database is limited in that it includes only information on patients diagnosed in 2000 or later. However, the SEER program began collecting information on metastases in patients who were diagnosed in 2010. Therefore, the fact that this database only included patients diagnosed since 2000 was not an issue for this study because it specifically required data on metastases. Consequently, only patients diagnosed in 2010 or later were included in the study cohort.

As described above, the first selection criterion for patients was diagnosis in 2010 or later. Other criteria for selecting patients were the primary site and histological classification of the tumor. Because the SEER program uses the International Classification of Diseases for Oncology (ICD-O) codes to classify the primary site and histological type of tumors and records them in the corresponding fields, the patient group for this study was selected using the following codes. The histological classification of the tumor was selected using the “ICD-O-3 Hist/behav” field, and the primary site of the tumor was selected using the “Site recode ICD-O-3/WHO 2008” field of the database. Based on the reporting criteria of the SEER program and the WHO classification, the most appropriate histological type was selected [7,33]. Specifically, code 8120/3 was used to select patients with conventional UC, code 8122/3 was used to select patients with sarcomatoid UC, code 8131/3 was used to select patients with micropapillary UC, code 8140/3 was used to select patients with ADC, code 8070/3 was used to select patients with SCC, and codes 8013/3, 8041/3, and 8246/3 were used to select patients with NEC. Patients with various histological tumor types were included in this study only if their tumors originated from the urinary bladder. However, for patients diagnosed with conventional UC, we also included those with tumors originating from the ureter and renal pelvis for comparison with those originating from the bladder. A flowchart summarizing the patient groups included in the statistical comparison at each stage, including the selection process, is shown in Figure S1.

For each selected patient, the extracted data included the year of diagnosis, ICD-O-3 histology codes, primary site, presence or absence of metastasis, and information on specific metastatic sites, including the bone, brain, liver, lung, distant lymph nodes (LNs), and other organs.

### 2.2. Definition of Organotropic Metastasis Rate

Typically, the metastasis rate is calculated as the percentage of patients with metastases divided by the total number of patients. Metastasis rates calculated in this manner are also valuable for comparing the propensity for metastasis between different patient populations. Similarly, the rate of metastasis to a specific organ can be calculated as the percentage of the number of patients with metastasis to a specific organ divided by the total number of patients. However, the metastasis rates to specific organs calculated in this manner cannot be used for comparisons between patient groups with different overall metastasis rates. This is because differences in overall metastasis rates introduce statistical errors. To address this issue, the rate of metastasis to a specific organ was calculated as the percentage of the number of patients with metastasis to a specific organ divided by the total number of patients with metastasis. This value, hereafter referred to as the “organotropic metastasis rate,” was used to make comparisons between different patient groups. This method has been used in several previous studies [21,22,34].

### 2.3. Statistical Analysis

Categorical variables such as the presence or absence of metastasis to a corresponding organ were compared using Pearson’s chi-square test or Fisher’s exact test, as appropriate. Statistical significance was defined as a two-tailed *p*-value less than 0.05. Statistical analyses were performed using SPSS (version 17.0; SPSS Inc., Chicago, IL, USA) and OriginPro (version 2023b; OriginLab, Northampton, MA, USA).

## 3. Results

### 3.1. Comparison of Metastatic Behavior in Different Histological Types of Bladder Cancer

A total of 48,789 patients with conventional UC, 1683 with NEC, 1667 with SCC, and 1003 with ADC were identified and retrieved from the database. The overall metastasis rate (percentage of patients with metastasis/total number of patients) of conventional UC was 8.8% (4317/48,789), which was significantly (all *p* < 0.001) lower than that of SCC (15.7%, 262/1667), ADC (18.4%, 185/1003), and NEC (26.0%, 438/1683). No significant difference (*p* = 0.067) in the overall metastasis rate was observed between the patients with SCC and those with ADC. The overall metastasis rate of patients with NEC was the highest among the four histological types and was significantly (all *p* < 0.001) higher than that of patients with conventional UC, SCC, or ADC.

As expected, statistically significant differences in overall metastasis rates were observed among the four histologically distinct tumor types. Such differences in the overall metastasis rates are likely to introduce statistical errors when comparing the tendency to metastasize to each specific organ. To eliminate the possibility of statistical errors and make accurate comparisons, organotropic metastasis rates were calculated as described in the Methods and were used to compare the propensity to metastasize to each specific organ.

**Figure 1:**
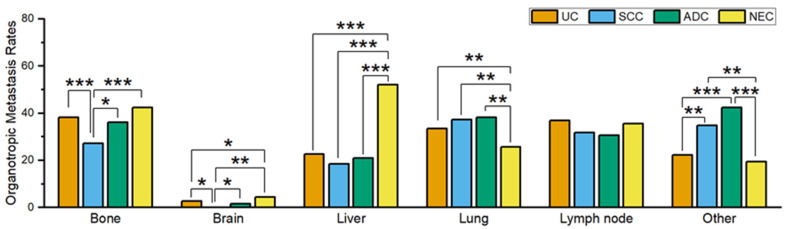
Comparison of organotropic metastasis rates for each organ across the four histologically distinct carcinomas. Statistically significant comparisons are marked with asterisks (* *p* < 0.05; ** *p* < 0.01; *** *p* < 0.001). Abbreviations: UC, urothelial carcinoma; SCC, squamous cell carcinoma; ADC, adenocarcinoma; NEC, neuroendocrine carcinoma.

Organotropic metastasis rates for each of the four histological tumor types and their comparisons are summarized in Table 1 and Figure 1. The bone organotropic metastasis rate in patients with SCC (27.2%, 68/250) was significantly lower than that in patients with conventional UC (38.3%, 1608/4194), patients with ADC (36.1%, 65/180), and patients with NEC (42.3%, 180/426) (*p* < 0.001, *p* = 0.049, and *p* < 0.001, respectively). Similarly, the brain organotropic metastasis rate in patients with SCC (0%, 0/251) was also significantly lower than that in patients with conventional UC (2.6%, 109/4175), patients with ADC (1.7%, 3/174), and patients with NEC (4.5%, 19/422) (*p* = 0.01, *p* = 0.037, and *p* = 0.001, respectively). In contrast, patients with NEC had a significantly (*p* = 0.024) higher brain organotropic metastasis rate (4.5%, 19/422) than those with conventional UC (2.6%, 109/4175). Patients with NEC also showed significantly (all *p* < 0.001) higher liver organotropic metastasis rates (52.1%, 223/428) than those with conventional UC (22.6%, 948/4190), SCC (18.4%, 46/250), or ADC (20.9%, 37/177). Lung organotropic metastasis rate was significantly lower in patients with NEC (25.7%, 109/424) than in those with conventional UC (33.5%, 1399/4181), SCC (37.2%, 93/250), or ADC (38.3%, 67/175) (*p* = 0.001, *p* = 0.002, and *p* = 0.002, respectively). There was no significant difference in the LN organotrophic metastasis rates among patients with conventional UC, SCC, ADC, and NEC. Interestingly, the rate of organotropic metastasis to other organs was lower in patients with UC or NEC, and higher in those with SCC or ADC. Specifically, the organotropic metastasis rate to other organs in patients with UC (22.2%, 481/2166) was not significantly (*p* = 0.364) different compared to that in patients with NEC (19.5%, 43/220), but was significantly lower than that in patients with SCC (34.8%, 46/132) and ADC (42.3%, 41/97) (*p* = 0.001 and *p* < 0.001, respectively). Similarly, the organotropic metastasis rate to other organs in patients with NEC (19.5%, 43/220) was significantly lower than that in SCC or ADC patients (*p* = 0.001 and *p* < 0.001, respectively). No significant (*p* = 0.253) difference was found in the organotropic metastasis rate to other organs between patients with SCC and ADC.

### 3.2. Comparison of Metastatic Behavior in Different Histological Subtypes of Bladder Primary UC

**Figure 2:**
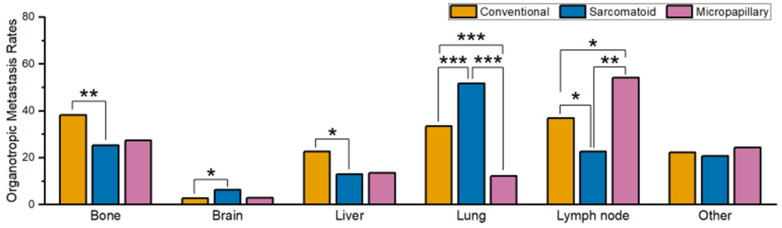
Comparison of organotropic metastasis rates for each organ in conventional urothelial carcinoma (UC), micropapillary UC, and sarcomatoid UC originating from the bladder. Statistically significant comparisons are marked with asterisks (* *p* < 0.05; ** *p* < 0.01; *** *p* < 0.001).

Of the 13 histological subtypes of UC currently accepted by the WHO classification, only giant cell UC, lymphoepithelioma-like UC, micropapillary UC, sarcomatoid UC, and poorly differentiated UC have been recorded separately in the SEER database [33]. The remaining histological subtypes were recorded together and included under the same ICD-O-3 code as conventional UC. Among the histological subtypes separately recorded in the SEER database, only 17 cases of giant cell UC (8 cases with metastasis), 88 cases of lymphoepithelioma-like UC (5 cases with metastasis), and 35 cases of poorly differentiated UC (9 cases with metastasis) were available from the SEER database. The number of patients included was considered insufficient for statistical analysis; therefore, they were excluded from further analysis. In contrast, 627 cases of micropapillary UC (66 cases with metastasis) and 675 cases of sarcomatoid UC (97 cases with metastasis) were retrieved from the SEER database. As the number of cases collected was sufficient to compare the metastatic patterns with those of conventional UC, a comparative analysis was performed for these two histological subtypes. A summary of the results of this analysis is presented in Table 2 and Figure 2.

The overall metastasis rate (percentage of patients with metastasis/total number of patients) of sarcomatoid UC patients was 14.4% (97/675), which was significantly higher than that of micropapillary UC patients (10.5%, 66/627) and conventional UC patients (8.8%, 4317/48,789) (*p* = 0.036 and *p* < 0.001, respectively). Patients with micropapillary UC also showed significantly (*p* < 0.001) higher overall metastatic rates than those with conventional UC. Significant differences in metastatic rates were observed among patients with conventional, sarcomatoid, and micropapillary UC. Therefore, as previously de-scribed, organotropic metastasis rates were calculated and compared to determine the tendency of metastasis to each specific organ.

Bone organotropic metastasis rate in patients with sarcomatoid UC (25.3%, 24/95) was significantly (*p* = 0.009) lower than that in patients with conventional UC (38.3%, 1608/4194). Similarly, the liver organotropic metastasis rate in patients with sarcomatoid UC (12.9%, 12/93) was significantly (*p* = 0.026) lower than in patients with conventional UC (22.6%, 948/4190). In contrast, the brain organotropic metastasis rate in patients with sarcomatoid UC (6.3%, 6/95) was significantly (*p* = 0.042) higher than in patients with conventional UC (2.6%, 109/4175). The analysis of organotropic metastasis rates to the lungs and LN revealed significant differences between conventional UC patients, sarcomatoid UC patients, and micropapillary UC patients, as well as between sarcomatoid UC patients and micropapillary UC patients. The lung organotropic metastasis rate was significantly (all *p* < 0.001) higher in sarcomatoid UC patients (51.6%, 47/91) compared to both conventional (33.5%, 1399/4181) and micropapillary (12.1%, 8/66) UC patients. The lowest lung organotropic metastasis rate was observed in micropapillary UC patients, which was also significantly (*p* < 0.001) lower compared to conventional UC patients. In contrast, micropapillary UC patients had a significantly higher LN organotropic metastasis rate (54.1%, 20/37) than that of conventional UC patients (36.8%, 792/2153) and sarcomatoid UC patients (22.6%, 12/53) (*p* = 0.031 and *p* = 0.002, respectively). Moreover, the LN organotropic metastasis rate in sarcomatoid UC patients was the lowest of the three groups and was also significantly (*p* = 0.035) lower than that of conventional UC patients. Finally, the organotropic metastasis rate to other organs did not differ significantly among the three histological subtypes.

### 3.3. Comparison of Metastatic Behavior in Patients with Conventional UC of Bladder, Ureter, and Renal Pelvis Primary

In addition to the previously retrieved 48,789 patients with bladder primary conventional UC, 4993 patients with renal pelvis primary conventional UC, and 2659 patients with ureter primary conventional UC were retrieved from the SEER database.

**Figure 3:**
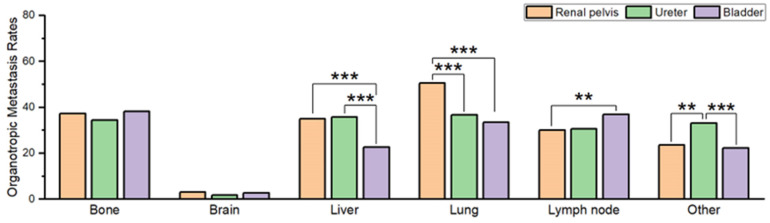
Comparison of organotropic metastasis rates in each organ in patients with conventional urothelial carcinoma originating from the bladder, ureter, or kidney. Statistically significant comparisons are marked with asterisks (** *p* < 0.01; *** *p* < 0.001).

Interestingly, the overall metastasis rates varied significantly according to the primary site, while comparisons were made between tumors of the same histological type. Patients with conventional UC of renal pelvis origin had an overall metastasis rate of 28.8% (1438/4993), which was significantly (all *p* < 0.001) higher than those with conventional UC of bladder (8.8%, 4317/48,789) or ureteral (15.6%, 414/2659) origins. The overall metastasis rate in patients with conventional UC of ureteral origin was also significantly (*p* < 0.001) higher than that in patients with conventional UC of bladder origin. As described in the previous section, organotropic metastasis rates were calculated and compared to determine whether there were any differences based on the origin of the tumor. The results are summarized in Table 3 and Figure 3.

No significant differences were observed in the rates of organotropic metastasis to the bone or brain based on the primary organ in conventional UC. However, there have been some observations of significant differences in metastatic behavior to specific organs according to the primary organ in conventional UC. The liver organotropic metastasis rate in patients with primary bladder UC (22.6%, 948/4190) was significantly (both *p* < 0.001) lower than in patients with primary ureter (35.8%, 144/402) or primary renal pelvis (35.0%, 491/1404) UC. The lung organotropic metastasis rate in patients with renal pelvic primary conventional UC (50.5%, 705/1396) was significantly (both *p* < 0.001) lower than that in patients with primary ureteral (36.7%, 148/403) or bladder primary (33.5%, 1399/4181) UC. The LN organotropic metastasis rate in patients with primary bladder UC (36.8%, 792/2153) was significantly (*p* = 0.001) higher than that in patients with primary renal pelvic UC (30.0%, 207/689). Finally, the organotropic metastasis rate to other organs was significantly higher in patients with primary ureter UC (33.0%, 69/209) than in those with primary renal pelvic (23.6%, 164/696) or bladder (22.2%, 481/2166) UC (*p* = 0.006 and *p* < 0.001, respectively).

### 3.4. Comparison of Metastatic Behavior between Conventional UC Patients Originating from Different Regions of the Bladder

A total of 48,789 patients diagnosed with conventional UC originating in the bladder were subcategorized based on the bladder region where the tumor originated. Most tumors originated from the lateral wall (8056 cases), followed by 4089 cases from the posterior wall, 2977 from the trigone, 2080 from the dome, 1591 from the neck, 1362 from the anterior wall, and ureteric orifice (889 cases).

**Figure 4:**
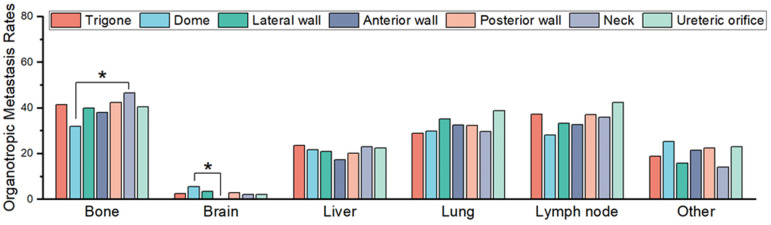
Comparison of organotropic metastasis rates for each organ in patients with conventional urothelial carcinoma originating from various bladder regions Statistically significant comparisons are marked with asterisks (* *p* < 0.05).

The overall metastatic rate varied depending on the primary tumor region. Conventional UCs originating from the neck had the highest overall metastasis rate at 9.4% (149/1591), followed by 8.2% (245/2977) from the trigone, 6.9% (94/1362) from the anterior wall, 6.0% (125/2080) from the dome, 5.9% (240/4089) from the posterior wall, and 5.8% (52/889) from the ureteral orifice. Tumors from the lateral wall had the lowest metastasis rate at 5.4% (437/8056). The organotropic metastasis rate was calculated and compared for each primary site. The results are summarized in Figure 4 and Table S1.

As expected, differences in the organotropic metastasis rates based on primary site were rarely significant. Only two comparisons showed statistically significant differences: the bone organotropic metastasis rate in patients with conventional neck primary UCs (46.6%, 68/146) was significantly (*p* = 0.015) higher than that in patients with dome-primary conventional UCs (32.0%, 40/125), and the brain organotropic metastasis rate in patients with dome-primary conventional UCs (5.6%, 7/125) was significantly (*p* = 0.021) higher than that in patients with anterior wall primary conventional UCs (0%, 0/93).

## 4. Discussion

Metastasis is the leading cause of death in patients with advanced cancer [13,35,36]. Therefore, early detection of metastases and proper treatment are crucial for improving the survival rate of patients with cancer. Research on how to effectively monitor cancer patients for the early detection of metastases with limited resources has long been of interest. If clinicians could predict in advance which organs are more likely to develop metastatic lesions, it would greatly help them plan a patient’s treatment and follow-up care. In this study, we examined the impact of tumor histology and primary site location on metastatic behavior. Since the histological type and primary site of the tumor are readily obtainable in current clinical practice without the introduction of new tests, the findings of this study are expected to be useful in clinical practice.

Significant differences in the organotropic metastasis rates were observed among UC, SCC, ADC, and NEC, which are the most common tumors encountered in bladder cancer. Although no prior studies have directly compared these metastatic patterns in bladder cancer, the high liver metastatic tendency and low lung metastatic tendency observed in NEC, as well as the low bone and brain metastatic tendencies in SCC, align with findings from previous studies conducted in different organs [21,22,34]. A comparison of organotropic metastatic tendencies to other organs showed interesting results. Based on these tendencies, the four tumor types were divided into two categories: SCC and ADC, which exhibited high organotropic metastatic tendencies to other organs, and UC and NEC, which displayed low organotropic metastatic tendencies to other organs. Unfortunately, in the SEER database, metastases to all organs, except the bone, brain, lungs, liver, and LNs, are lumped together and recorded as metastases to other organs. Therefore, as this study relied on the SEER database, it was not possible to analyze the differences in organotropic metastatic tendencies to other organs in more detail, which is a limitation of this study. Another limitation of this study was the inability to compare UC with divergent differentiation patterns, such as squamous, glandular, and neuroendocrine differentiation, with other tumor types. Squamous differentiation is the most common divergent differentiation observed in UC, followed by glandular differentiation; although rare, neuroendocrine differentiation has also been documented [7,37]. It would have been very interesting to compare the metastatic tendencies of pure conventional UC and UC with divergent differentiation, such as squamous, glandular, and neuroendocrine, as well as pure SCC, ADC, and NEC. Unfortunately, such comparisons were not possible in this study based on the SEER database, and further research is expected to be conducted in the future.

Although it was expected that there would be differences in organotropic metastatic tendencies between tumors with distinct histological characteristics, the finding of significant variations in organotropic metastatic tendencies within the same UC according to histological subtype is interesting. In this study, we compared only two histological subtypes, micropapillary and sarcomatoid, with conventional UC. However, both histological subtypes are associated with poor prognosis, warranting special attention; thus, the significance of this study is substantial [38,39]. The overall metastasis rate in patients with sarcomatoid UC or micropapillary UC was significantly higher than that in patients with conventional UC, supporting previous reports that sarcomatoid UC and micropapillary UC have a worse prognosis than conventional UC.

When comparing the metastatic tendencies of the three histological subtypes, the most interesting finding was that the contrasting tendencies for organotropic metastasis displayed by the micropapillary and sarcomatoid subtypes, primarily in the lungs and LNs. The micropapillary subtype showed a high prevalence of LN-specific organotropic metastases, but a low prevalence of lung-specific organotropic metastases. In contrast, the sarcomatoid subtype showed a high prevalence of lung-specific organotropic metastases but a low prevalence of LN-specific organotropic metastases. The high rate of LN-specific organotropic metastasis in the micropapillary subtype is probably due to the frequent occurrence of lymphovascular invasion in this subtype compared with other histological subtypes [39]. Furthermore, a high lung-specific organotropic metastasis rate observed in tumors displaying sarcomatoid differentiation was documented in a study focusing on renal cell carcinoma. However, the interesting observations made in this study still pose challenges for adequate explanations through existing research and require further investigation.

In the present study, we observed significant differences in organotropic metastatic tendencies depending on the primary site, even within the same conventional UC. Anatomical variations in the venous drainage of each organ should be considered to explain this difference. However, while the renal pelvis, ureter, and bladder may have different intermediate pathways, their venous blood ultimately drains into the inferior vena cava. Therefore, anatomical differences alone may not suffice to explain variations in metastatic tendencies.

Previous studies have reported that UC originating in the upper urinary tract, such as the renal pelvis and ureter, exhibits distinct molecular genetic characteristics from UC originating in the bladder [27,29]. It is plausible that differences in molecular genetic features may contribute to variations in organotropic metastatic tendencies. In-depth research on the molecular genetic characteristics of UCs arising from the renal pelvis, ureter, and bladder, their disparities, and their associations with metastatic tendencies is needed.

Distinct organotropic metastatic tendencies were not observed in conventional UCs arising from different regions of the bladder. This was a reasonably anticipated outcome. However, significant differences were noted in the comparison of bone organotropic metastasis rates between tumors originating in the neck and dome, or brain organotropic metastasis rates between tumors originating in the anterior wall and dome. Nevertheless, these results should be interpreted with caution. Although there is the possibility of true significance, it is likely that there are discrepancies from real-world observations and that these statistically significant differences are limited to the scope of this study. However, further research is required to confirm these findings.

As previously mentioned, this study has encountered several limitations. Firstly, there are limitations that arise from the fact that this study uses only information recorded in the SEER database, which have already been discussed previously. Additionally, irrespective of the limitations associated with the SEER database, it is worth noting that this study focused exclusively on analyzing metastatic patterns based on histological diagnosis and the primary site of the tumor. Bladder cancer is known to be influenced by various factors affecting prognosis, with new factors continuously emerging through recent research. For instance, a recent study has reported that female bladder cancer patients, not only those diagnosed with urothelial carcinoma but also those with non-urothelial variant histology, tend to be diagnosed at more advanced stages and exhibit higher cancer-specific mortality rates [5]. However, gender-related differences in metastatic tendencies were not addressed in this study. Lastly, while this study demonstrated differences in metastatic patterns based on histological diagnosis and primary tumor site, it did not delve into the reasons behind these differences.

Despite these limitations, the results of this study are expected to provide valuable insights for clinical practice. This research has revealed distinct disparities in metastatic tendencies based on histological diagnosis and primary tumor site. Such information can prove valuable for the surveillance of bladder cancer patients, particularly in the development of post-surgical follow-up plans. For example, in the case of patients diagnosed with NEC, not only do they exhibit a higher metastatic rate, but they also display a pronounced propensity for liver metastasis, making it imperative to prioritize monitoring for liver metastasis when devising follow-up plans compared to patients with different histological diagnoses. Furthermore, the limitations outlined earlier in this study could serve as promising avenues for future research. It is hoped that further investigations will uncover the underlying causes of these divergent metastatic patterns.

## 5. Conclusions

This study demonstrated distinct organotropic metastatic tendencies among the major tumor types of bladder cancer, namely, UC, SCC, ADC, and NEC. Additionally, UC exhibits varying organotropic metastatic tendencies based on the histological subtype and primary site. Histological subtypes and primary tumor sites provide readily accessible clinical information and require no additional testing. The results of this study may have value in the development of patient surveillance plans for the early detection of metastatic disease. Further in-depth research is warranted to elucidate the mechanisms underlying the differences observed in the histological patterns and primary site-related variations identified in this study.

## Figures and Tables

**Table 1:** Summary of organotropic metastasis rates in each of the four histologically distinct bladder primary carcinomas.

	UC	SCC	ADC	NEC
Overall Metastasis Rates (Pts with metastasis/total number of Pts)				
	8.8% (4317/48,789)	15.7% (262/1667)	18.4% (185/1003)	26.0% (438/1683)
Organotropic Metastasis Rates (Pts with metastasis to the indicated organ/Pts with metastasis)				
Bone	38.3% (1608/4194)	27.2% (68/250)	36.1% (65/180)	42.3% (180/426)
Brain	2.6% (109/4175)	0% (0/251)	1.7% (3/174)	4.5% (19/422)
Liver	22.6% (948/4190)	18.4% (46/250)	20.9% (37/177)	52.1% (223/428)
Lung	33.5% (1399/4181)	37.2% (93/250)	38.3% (67/175)	25.7% (109/424)
LN	36.8% (792/2153)	31.8% (42/132)	30.6% (30/98)	35.5% (78/220)
Other	22.2% (481/2166)	34.8% (46/132)	42.3% (41/97)	19.5% (43/220)

UC, urothelial carcinoma; SCC, squamous cell carcinoma; ADC, adenocarcinoma; NEC, neuroendocrine carcinoma; Pts, patients; LN, lymph node.

**Table 2:** Summary of organotropic metastasis rates in conventional urothelial carcinoma (UC), micropapillary UC, and sarcomatoid UC originating from the bladder.

	Conventional UC	Sarcomatoid UC	Micropapillary UC
Overall Metastasis Rates (Pts with metastasis/total number of Pts)			
	8.8% (4317/48,789)	14.4% (97/675)	10.5% (66/627)
Organotropic Metastasis Rates (Pts with metastasis to the indicated organ/Pts with metastasis)			
Bone	38.3% (1608/4194)	25.3% (24/95)	27.3% (18/66)
Brain	2.6% (109/4175)	6.3% (6/95)	3.0% (2/66)
Liver	22.6% (948/4190)	12.9% (12/93)	13.6% (9/66)
Lung	33.5% (1399/4181)	51.6% (47/91)	12.1% (8/66)
LN	36.8% (792/2153)	22.6% (12/53)	54.1% (20/37)
Other	22.2% (481/2166)	20.8% (11/53)	24.3% (9/37)

UC, urothelial carcinoma; Pts, patients; LN, lymph node.

**Table 3:** Summary of organotropic metastasis rates in patients with conventional urothelial carcinomas originating from the bladder, ureter, or renal pelvis.

	Renal Pelvis	Ureter	Bladder
Overall Metastasis Rates (Patients with metastasis/total number of patients)			
	28.8% (1438/4993)	15.6% (414/2659)	8.8% (4317/48,789)
Organotropic Metastasis Rates (Patients with metastasis to the indicated organ/Patients with metastasis)			
Bone	37.3% (522/1401)	34.4% (139/404)	38.3% (1608/4194)
Brain	3.1% (43/1383)	1.8% (7/398)	2.6% (109/4175)
Liver	35.0% (491/1404)	35.8% (144/402)	22.6% (948/4190)
Lung	50.5% (705/1396)	36.7% (148/403)	33.5% (1399/4181)
Lymph node	30.0% (207/689)	30.6% (63/206)	36.8% (792/2153)
Other	23.6% (164/696)	33.0% (69/209)	22.2% (481/2166)

## Data Availability

The data presented in this study are available on request from the corresponding author.

## Supplementary Materials

The following supporting information can be downloaded at: https://www.mdpi.com/article/10.3390/curroncol30100656/s1, Table S1: Summary of organotropic metastasis rates in patients with conventional urothelial carcinoma originating from various regions of the bladder; Figure S1: A flowchart summarizing the patient groups included in the statistical comparison at each stage, including the selection process.
