# Supplementary material for: Do Histology and Primary Tumor Location Influence Metastatic Patterns in Bladder Cancer?
Source: Curr Oncol. 2023 Oct 11;30(10):9078–89. doi: 10.3390/curroncol30100656 (PMC10605465; doi:10.3390/curroncol30100656)

Comparison of metastatic behavior in different histological types of bladder cancer

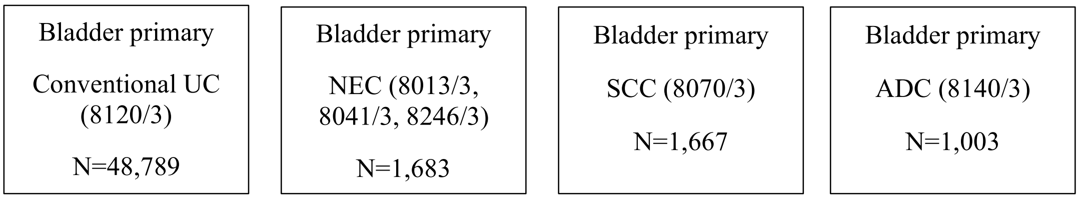

Comparison of metastatic behavior in different histologic subtypes of bladder primary UC

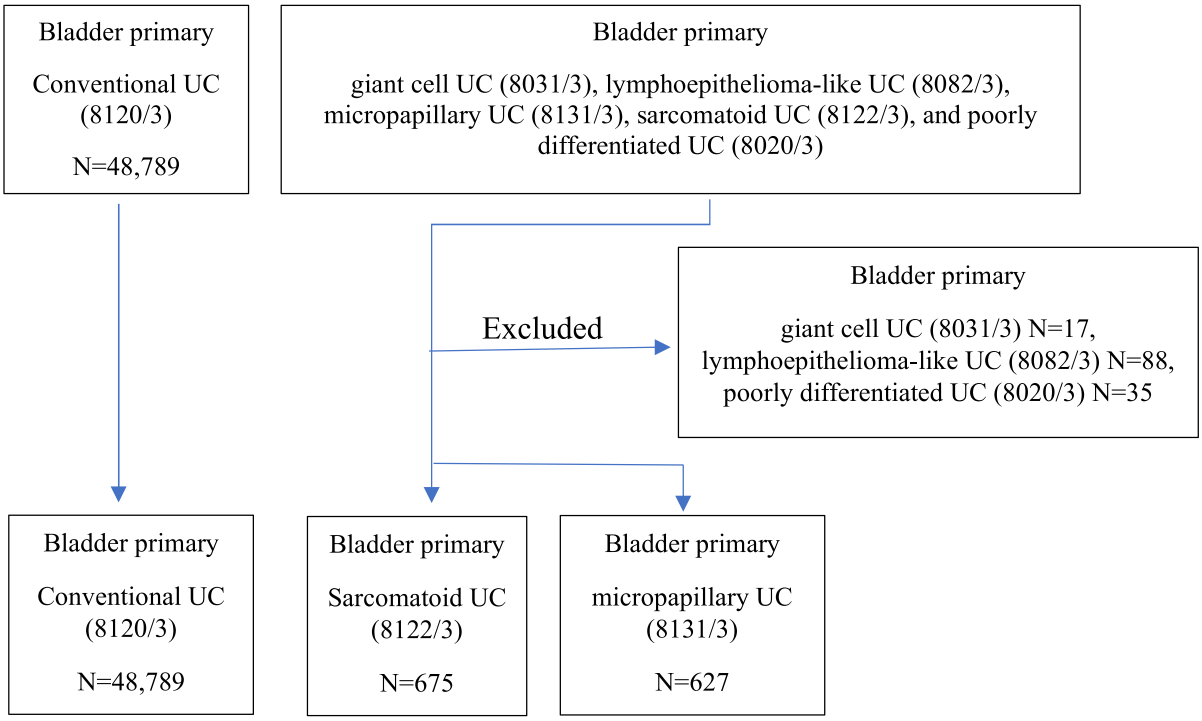

Comparison of metastatic behavior in patients with conventional UC of bladder, ureter, and renal pelvis primary

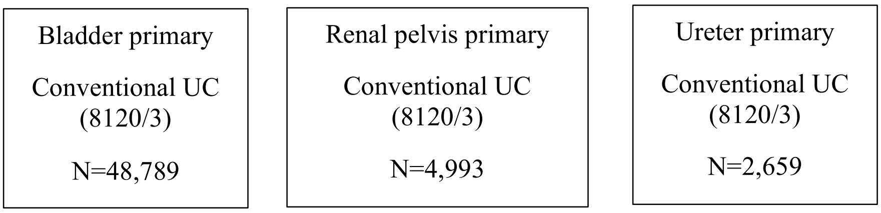

Comparison of metastatic behavior between conventional UC patients originating from different regions of the bladder

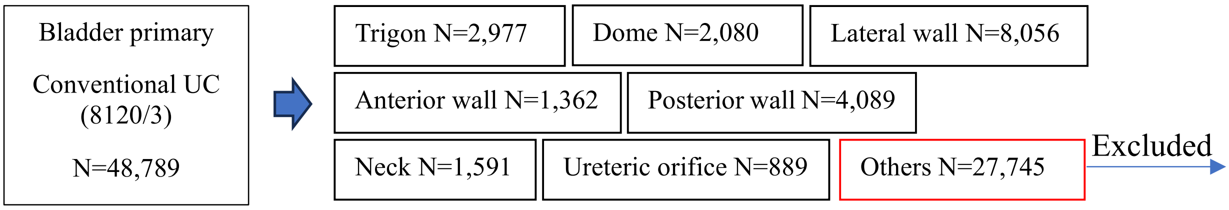

Supplement: Supplementary file 1 [file curroncol-30-00656-s001.zip › Figure S1.pdf]
