# Supplementary material for: Do Histology and Primary Tumor Location Influence Metastatic Patterns in Bladder Cancer?
Source: Curr Oncol. 2023 Oct 11;30(10):9078–89. doi: 10.3390/curroncol30100656 (PMC10605465; doi:10.3390/curroncol30100656)
Supplement: Supplementary file 1 [file curroncol-30-00656-s001.zip › Table S1.pdf]

Table S1. Summary of the organotropic metastasis rates in patients with conventional urothelial carcinoma originating from various regions in the bladder.

	Trigone	Dome	Lateral wall	Anterior wall	Posterior wall	Neck	Ureteric orifice
Overall Metastasis Rates (Patients with metastasis/total number of patients)							
	8.2% (245/2977)	6.0% (125/2080)	5.4% (437/8056)	6.9% (94/1362)	5.9% (240/4089)	9.4% (149/1591)	5.8% (52/889)
Organotropic Metastasis Rates<br>(Patients with metastasis to the indicated organ/Patients with metastasis)							
Bone	41.4% (101/244)	32.0% (40/125)	40.0% (171/427)	38.0% (35/92)	42.4% (101/238)	46.6% (68/146)	40.4% (19/47)
Brain	2.5% (6/243)	5.6% (7/125)	3.5% (15/428)	0% (0/93)	3.0% (7/237)	2.1% (3/145)	2.1% (1/48)
Liver	23.6% (57/242)	21.8% (27/124)	20.9% (89/426)	17.4% (16/92)	20.2% (48/238)	23.1% (34/147)	22.4% (11/49)
Lung	28.9% (70/242)	29.8% (37/124)	35.1% (150/427)	32.6% (30/92)	32.3% (76/235)	29.7% (43/145)	38.8% (19/49)
Lymph node	37.2% (55/148)	28.1% (18/64)	33.2% (76/229)	32.7% (18/55)	37.1% (43/116)	35.9% (23/64)	42.3% (11/26)
Other	18.9% (28/148)	25.4% (16/63)	15.7% (36/229)	21.4% (12/56)	22.4% (26/116)	14.1% (9/64)	23.1% (6/26)
